# Endogenous Enzymatic Activity in Dentin Treated with a Chitosan Primer

**DOI:** 10.3390/ijms22168852

**Published:** 2021-08-17

**Authors:** Tatjana Maravić, Eugenia Baena, Claudia Mazzitelli, Uroš Josić, Edoardo Mancuso, Vittorio Checchi, Luigi Generali, Laura Ceballos, Lorenzo Breschi, Annalisa Mazzoni

**Affiliations:** 1 Department of Biomedical and Neuromotor Sciences, DIBINEM, University of Bologna-Alma Mater Studiorum, Via San Vitale 59, 40125 Bologna, Italy; tatjana.maravic@unibo.it (T.M.); claudia.mazzitelli@unibo.it (C.M.); uros.josic2@unibo.it (U.J.); edoardo.mancuso2@unibo.it (E.M.); lorenzo.breschi@unibo.it (L.B.); 2 Area of Stomatology, Health Sciences Faculty, King Juan Carlos University, Avda. de Atenas, 28922 Alcorcón, Spain; eugenia.baena@urjc.es (E.B.); laura.ceballos@urjc.es (L.C.); 3 Department of Surgery, Medicine, Dentistry and Morphological Sciences with Transplant Surgery, Oncology and Regenerative Medicine Relevance, University of Modena & Reggio Emilia, 41124 Modena, Italy; vittorio.checchi@unimore.it (V.C.); luigi.generali@unimore.it (L.G.)

**Keywords:** dentin, chitosan, collagen cross-linker, matrix metalloproteinases, zymography

## Abstract

The aim of this study was to evaluate the effect of different concentrations of chitosan polymer on dentinal enzymatic activity by means of gelatin and in situ zymography. Human dentin was frozen and ground in a miller. Dentin powder aliquots were demineralized with phosphoric acid and treated with three different concentrations of lyophilized chitosan polymer (1, 0.5 and 0.1 wt%) dissolved in distilled water. Dentin proteins were extracted from each experimental group and electrophoresed under non-reducing conditions in 10% SDS-PAGE containing fluorescein-labeled gelatin. After 48 h in the incubation buffer at 37 °C, proteolytic activity was registered under long-wave UV light scanner and quantified by using Image J software. Furthermore, additional teeth (*n* = 4) were prepared for the in situ zymographic analysis in unrestored as well as restored dentin pretreated with the same chitosan primers. The registered enzymatic activity was directly proportional to the chitosan concentration and higher in the restored dentin groups (*p* < 0.05), except for the 0.1% chitosan primer. Chitosan 0.1% only showed faint expression of enzymatic activity compared to 1% and 0.5% concentrations. Chitosan 0.1% dissolved in water can produce significant reduction in MMPs activity and could possibly contribute to bond strength preservation over time.

## 1. Introduction

Dentin bonding of composite restoration implies the complete or partial demineralization of the dentin surface according to the adhesive strategy employed, etch-and-rinse or self-etch. The collagen matrix is then infiltrated with resin monomers and the hybrid layer is established [1]. However, resin monomers are not able to fully infiltrate the collagen network, and the presence of denuded collagen fibrils is unavoidable making the hybrid layer susceptible to hydrolytic degradation promoted by endogenous dentinal proteases [1,2].

These enzymes play an important role in the hydrolysis of collagen fibrils within hybrid layers, causing the loss of bond strength between the tooth-colored restoration and the underlying mineralized dentin over time [1,2,3]. Matrix-metalloproteinases (MMPs) and cysteine cathepsins are latent in mineralized dentin but are activated during dentin bonding procedures by etching, as well as both by etch-and-rinse and self-etch adhesives [4,5]. In fact, intense MMP-2 and MMP-9 activities have been detected at the basal part of the hybrid layer [6]. 

The recent literature suggests different strategies as an attempt to minimize or delay the hybrid layer degradation by endogenous proteases and, consequently, increase the longevity of the bonded restorations. There are two main trends under investigation, MMPs inhibitors and cross-linking agents [5,7,8]. The natural polymer chitosan has also been reported among the cross-linkers because of its ability to form a micro and nano-fibrillar collagen–chitosan network of superior mechanical properties [9]. This polymer is also bioadhesive, a desirable property for increasing the retention at the site of application [10]. 

Regarding chitosan and dentin bonding, the literature is scarce [11,12,13]. Recent findings showed promising results when chitosan was incorporated within an adhesive or modified with methacrylates in terms of microtensile bond strength, antibacterial properties [12,14] and protection of the adhesive–dentin interface after thermo-mechanical cycling [11]. Recently, there has been evidence of a potential anti-enzymatic effect of chitosan [15,16]. Nevertheless, literature has not evaluated whether the influence of chitosan on the dentinal enzymatic activity is dose-dependent, which is necessary in order to understand the underlying complex interaction of this natural cross-linker with the constituents of the extracellular dentinal matrix, as well as its interaction with the restorative materials.

Accordingly, the aim of the present study was to evaluate, by means of gelatin and in situ zymography, the effect of different concentrations of lyophilized chitosan polymer as a separate aqueous primer on the enzymatic activity of unrestored, as well as adhesively restored, dentin. The null hypotheses to be tested include the following: (1) chitosan applied on acid-etched dentin does not influence MMPs activity regardless of the concentration used; (2) the application of the adhesive resin does not influence the interaction of chitosan with the endogenous dentinal enzymes.

## 2. Results

### 2.1. Gelatin Zymography

**Figure 1:**
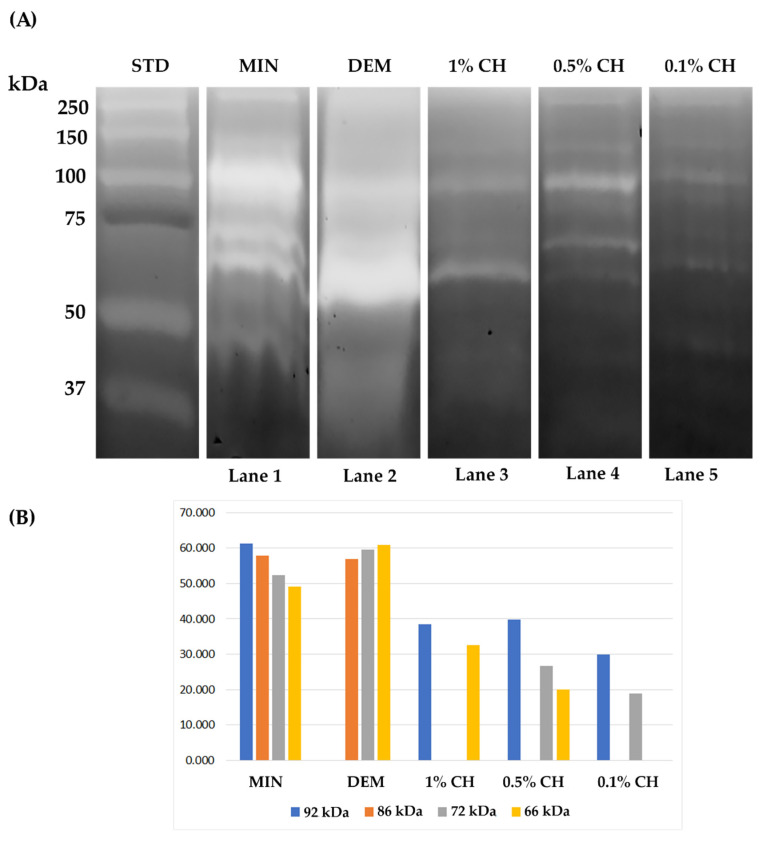
Zymographic analysis of proteins extracted from dentin powder. (**A**) Figure showing the differences in the enzymatic expression and activity between the investigated groups presented as light bands in the area of the molecular weights of MMP-2 and MMP-9. (**B**) Densitometric evaluation of the bands in the different treatment groups.

Results of the gelatin zymography are shown in Figure 1. Proteins extracted from mineralized dentin powder (Lane 1) showed the expression of pro-MMP-9 with the corresponding molecular weight of 92 kDa and active form of MMP-9 (at 86 kDa). Moreover, the presence of pro-form and active forms of MMP-2 is evident (72 and 66 kDa, respectively). On the other hand, the demineralization of dentin powder with phosphoric acid (Lane 2) demonstrates an increase in the expression of MMPs, but particularly MMP-2 in pro-form and active forms. The bands are wide and less delineated.

A lower expression of pro-MMP-9 compared to the control groups was detectable at 1% (lane 3) and 0.5% (lane 4) chitosan groups, and the faintest band was noted at 0.1% CH group (Lane 5). Furthermore, the expression of the 72 kDa MMP-2 pro-form decreased or completely disappeared after treating demineralized dentin with any of the chitosan concentrations tested (1%, 0.5% or 0.1%). In the groups treated with chitosan 0.5% and 1%, there is a faint band at the molecular weight of MMP-2 active form.

### 2.2. In Situ Zymography

**Figure 2:**
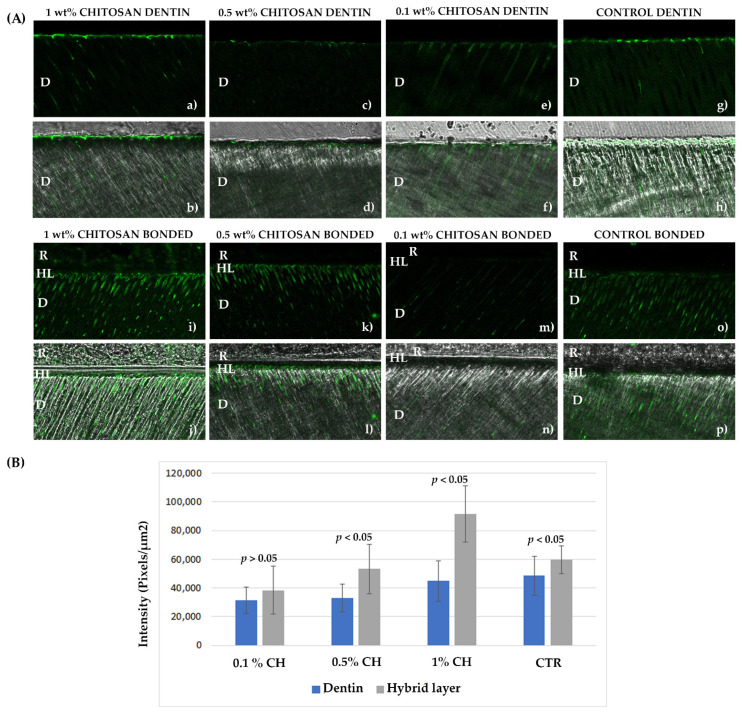
In situ zymography. (**A**) Images acquired in the green channel showing fluorescence within the dentin surface (**a**,**c**,**e**,**g**) and within the HL (**i**,**k**,**m**,**o**) of the tested groups. Images obtained by merging the differential interference contrast (DIC) image (showing optical density of the resin–dentin interface) and the image acquired in the green channel (**b**,**d**,**f**,**h**,**j**,**l**,**n**,**p**). (**B**) Quantification of the gelatinolytic activity within the dentin and the HL of the tested groups. D—dentin; HL—hybrid layer; R—resin composite.

The qualitative and quantitative results of the in situ zymography are presented on Figure 2. The gelatinolytic activity in unrestored dentin (Figure 2a–h) is concentrated mostly in the first several microns of the dentin surface (higher activity), as well as in the dentinal tubules (lower activity). The activity within the hybrid layer (Figure 2i–p) is visibly higher in all the groups compared to unrestored dentin, except for the 0.1% chitosan group. The high fluorescence level in the hybrid layers reaches deeper into the dentin tubules (~10 µm). The two-way ANOVA analysis of the quantitative data confirmed the qualitative observations and demonstrated that both investigated factors (“pretreatment” and “adhesive system application”), as well as their interaction, have a statistically significant influence on the gelatinolytic activity within dentin and the hybrid layer (*p* < 0.05). The intensity of the fluorescent signal significantly differed between all the pretreatments in the following order: 1% chitosan > control > 0.5% chitosan > 0.1% chitosan (*p* < 0.05). The application of the adhesive in all the pretreatment groups except for the 0.1% chitosan (*p* > 0.05) increased the density of the fluorescent signal (*p* < 0.05). Within the groups of unrestored dentin, significant differences were noted between all the groups except for the control and 1% chitosan, as well as 0.5 and 0.1% chitosan (control = 1% chitosan > 0.5% chitosan = 0.1% chitosan), while in the groups bonded with the adhesive and restored with composite resin, only the control group and the 0.5% groups were similar to one another (1% chitosan > control = 0.5% chitosan > 0.1% chitosan).

## 3. Discussion

The present study demonstrated that the inhibition of endogenous dentinal enzymes by lyophilized chitosan is dose dependent. The most efficient concentration of the aqueous chitosan primer in terms of MMPs inhibition is the lowest used dose, which is 0.1%. In view of the present results, the first null hypothesis has to be partially rejected. The silencing effect of chitosan on the endogenous enzymatic activity was hampered by the application of the adhesive resin and the composite restoration, except in the 0.1% chitosan-treated groups, resulting in the partial rejection also of the second null hypothesis.

Chitosan is a polysaccharide obtained from the de-N-acetylation of chitin which is the second most abundant biopolymer after cellulose. Chitin is mainly found in invertebrates such as crustacean shells or insect cuticles [17]. If not degraded, it is insoluble in neutral or basic pH conditions while soluble in acidic conditions (pH < 6), which may limit its applications [18]. The degradation of chitosan allows obtaining oligomers from the polymer [19] and facilitates its solubility in water, which may increase its application range. Its chemical structure has three types of reactive functional groups, an amino group and a primary and secondary hydroxyl group, which enable chemical modifications of chitosan in order to obtain derivatives for specific applications [18]. This natural polymer and cross-linker is characterized by interesting properties such as antibacterial activity, bio-adhesion and ability to be combined with a variety of biomaterials [18]. These desirable properties explain its increasing application in medical and dental areas [19,20,21]. 

In the past decade, there has been a great interest in the research community to develop strategies for enhancing the longevity of composite resin restorations and, consequently, decrease high global costs associated with their replacement [22]. One of the investigated strategies entails endogenous enzymes inhibition/inactivation and modification of the extracellular dentinal matrix [1,23]. On one hand, exogenous MMP inhibitors such as chlorhexidine, galardin, benzalkonium chloride or quaternary ammonium salts contain a functional group that can interact with the Zn^2+^ ion in the MMP molecule blocking its activation [1,24,25,26,27,28]. On the other hand, synthetic and natural cross-linkers such as genipin, carbodiimide (EDC) or grape seed extract form intermolecular and intramolecular collagen cross-links by means of stable covalent bonds, increasing the resin–dentin interface longevity [29,30,31,32]. Furthermore, cross-linkers have also been reported to decrease collagenolytic activity and inactivate MMPs [30,33,34]. This effect probably occurs due to conformational changes of the enzyme caused by the cross-linking of its (non)catalytic portion, impairing the ability of the enzyme to recognize and cleave the substrate [33,34,35]. The MMPs inactivation could be irreversible and, hence, permanent. The longest in vitro aging reported so far demonstrated the cross-linking effect after 5 years of in vitro aging [36]. Notwithstanding, it must be highlighted that cross-linkers do not behave as a group, as the enzymatic inactivation has been described to be cross-linker and time-dependent [30]. For instance, certain cross-linkers can form only intramolecular, while others can also form intermolecular cross-links. This depends on the length of the cross-linking molecule, which needs to be able to bridge around 1.3–1.7 nm in order to form intramolecular cross-links [37].

The data available on the cross-linking effect of chitosan are scarce but are in accordance with the data available on other cross-linkers. Previously, our research group demonstrated by means of gelatin zymography that the 0.1% chitosan primer was able to inhibit the expression of the MMPs when used with a universal adhesive in the SE mode. However, it activated the MMPs when used before a 3-step etch-and-rinse adhesive [15]. The results of the present study are in accordance with the previous findings as chitosan dissolved in water at low concentrations was able to decrease the activity and expression of dentinal endogenous proteases when used on unrestored dentin as well as before a universal adhesive placed in the etch-and-rinse mode. This indicates that the anti-enzymatic activity of chitosan is also adhesive system dependent. It seems that chitosan (0.1% water solution) is more effective when used with universal adhesives. Another interesting point to discuss is the influence of the application of the adhesive resin over a cross-linking primer. In the present study, the adhesive resins significantly increased the enzymatic activity in the dentin underlying the HL. It has been previously shown that both the etch-and-rinse and self-etch adhesives activate the dentinal MMPs [38,39,40], which is in accordance with the results of the present study (for all the groups except the 0.1% chitosan). Universal adhesives are the latest generation of adhesive systems containing functional monomers that enable them to create chemical bonds to the dental substrate, as well as to restorative materials (metal, composite and ceramics). These adhesives can be used in the etch-and-rinse, as well as in the self-etch mode [1]. They contain acidic monomers that simultaneously etch and infiltrate the dentin surface creating homogenous thin hybrid layers. However, these monomers can also activate the MMPs. 

It was observed in the present study that the chitosan concentration had an influence on the MPs expression. In this case, the lower the chitosan concentration, the lower the enzymatic activity. Chitosan 1% and 0.5% revealed gelatinolytic bands corresponding to active MMP-2, while there were no active forms of MMPs expressed in the 0.1% CH group, and the pro-form MMP-9 and MMP-2 bands were faint. In situ zymography confirmed these findings, with the 0.1% chitosan being the most efficient solution in terms of MMPs inhibition, both in unrestored and restored dentin. Elsaka et al. [14] also found that when incorporating chitosan within an adhesive resin, the concentration influenced the adhesive properties. The author concluded that the lowest concentration tested (0.12%) did not negatively interfere with the desirable adhesive resin characteristics; thus, it is expected that 0.1% chitosan behaves similarly. On the other hand, we found that the 1% chitosan primer either does not influence the enzymatic activity (in restored teeth) or provokes an undesirable effect of enzymatic activation (in non-restored dentin). In fact, the 1% chitosan primer used in the present study was more viscous compared to the lower concentration primers, which could have interfered with the wetting of the dentin and the penetration of the adhesive resin within the collagen network. Contrary to this, Neves et al. [16] demonstrated that the primers containing chitosan bound lipid nanoparticles are not efficient in the inhibition of dentinal enzymes in the concentrations of 0.4 and 0.6%, but only in a higher concentration of 2%. This discrepancy could be due to differences in the properties and behavior of chitosan bound to nanoparticles attributed to their nanometric scale [16].

The limitation of the present study could be that it was mainly focused on the anti-enzymatic properties of chitosan and did not investigate the mechanical properties of the chitosan-cross-linked collagen scaffold or of the hybrid layer with regards to different concentrations of the chitosan primer. However, an ongoing study of this research group aims to investigate these important topics. Moreover, future clinical studies should be performed with cross-linking agents, particularly the natural and non-toxic ones, in order to investigate the effectiveness of these compounds on the long-term clinical performance of direct and indirect adhesive restorations.

## 4. Materials and Methods

Reagents were purchased from Sigma Chemical (St. Louis, MO, USA) unless otherwise specified. 

### 4.1. Preparation of Solutions

Three aqueous solutions of 1, 0.5 and 0.1 wt% chitosan (Chitoscience Chitosan, Heppe Medical Chitosan GmbH, Halle, Germany; average molecular weight ~50 kDa) were obtained by dissolving the lyophilized polymer in distilled water without the need for pH adjustment. 

### 4.2. Gelatin Zymography

Ten sound human third molars were extracted after obtaining informed consent. The teeth were ground free of enamel, and pulpal tissue was completely removed. Dentin was frozen in liquid nitrogen and triturated to obtain dentin powder by using a Retsch miller (Model MM400, Retsch GmbH, Haan, Germany).

Mineralized (MD) dentin was divided into 5 experimental groups (two 100 mg aliquots per group) to be treated with 100 µL of previously prepared solutions:

- Group 1 (MIN): MD left untreated;
- Group 2 (DEM): MD was treated with 10 wt% phosphoric acid for 10 min at 4 °C, then the acid was neutralized with 4N NaOH and centrifuged;
- Group 3 (1% CH): MD was etched as in Group 3 and then treated with 1% chitosan water solution for 30 min;
- Group 4 (0.5% CH): MD was etched as in Group 3 and then treated with 0.5% chitosan water solution for 30 min;
- Group 5 (0.1% CH): MD was etched as in Group 3 and then mixed with 0.1% chitosan water solution for 30 min.

After the treatment, dentin aliquots were rinsed twice with distilled water and centrifuged to remove the supernatant. Dentin powder aliquots were stirred with the extraction buffer (50 mM Tris–HCl pH 6 containing 5 mM CaCl_2_, 100 mM NaCl, 0.1% Triton X-100, 0.1% nonionic detergent P-40 and 0.1 mM ZnCl_2_, 0.02% NaN_3_) for 24 h at 4 °C. Thereafter, the aliquots were sonicated for 10 min and then centrifuged twice in order to separate the supernatant (20 min/4 °C/12,000 rpm twice). The protein content was concentrated from the supernatant by using the Vivaspin centrifugal concentrator (10,000 kDa cut off; Vivaspin Sartorius Stedim Biotech, Goettingen, Germany) (30 min/4 °C/10,000 rpm for 3 times). The total protein concentration in the dentin extracts was determined by Bradford assay. 

Dentin proteins aliquots from each experimental group (60 μg) were diluted in a Laemmli sample buffer in a 4:1 ratio and electrophoresed under non-reducing conditions in 10% sodium dodecyl sulfate-polyacrylamide gel (SDS-PAGE) containing 1 mg/mL fluorescein-labeled gelatin. Pre-stained low-range molecular weight SDS-PAGE standards (Bio-Rad, Hercules, CA, USA) were used as molecular-weight markers. After electrophoresis, the gels were washed twice for 30 min in 2% Triton X-100 and incubated in zymography activation buffer (50 mmol/L Tris–HCl, 5 mmol/L CaCl_2_, pH 7.4) for 48 h at 37 °C. Gelatinase zymograms were registered under long-wave UV light scanner (ChemiDoc Universal Hood, Bio-Rad, Hercules, CA, USA). Zymograms were quantified by taking a mineralized group as baseline and using the Image J software (NIH, Bethesda, MD, USA).

### 4.3. In Situ Zymography

Sound extracted third molars (*n* = 5, sample size determined using G*Power 3.1.9.7 for Windows, Düsseldorf, Germany) [41] were used either within 24 h from the extraction or frozen immediately (−20 °C) until use. The enamel and superficial dentin were removed from all the teeth using a high-speed diamond saw with water cooling (Micromet, Remet, Bologna, Italy). When middle dentin was reached, two transversal cuts were made to obtain 1 mm thick slices of middle/deep dentin. These slices were further cut into 4 pieces, adding up to 8 dentin pieces per tooth. In order to test all the groups on the same dentin substrate, one dentin slice from each tooth was assigned to the 8 following groups:

- Group 1: Dentin etched with 37% phosphoric acid for 15 s (Vococid, Voco, Cuxhaven, Germany), rinsed thoroughly, blot dried and then primed with the 1 wt% chitosan water solution for 1 min, after which it was gently air-dried for 5 s;
- Group 2: Dentin etched and rinsed as in Group 1 and then primed with the 0.5 wt% chitosan water solution for 1 min, after which it was gently air-dried for 5 s;
- Group 3: Dentin etched and rinsed as in Group 1 and then primed with the 0.1 wt% chitosan water solution for 1 min, after which it was gently air-dried for 5 s;
- Group 4 (control): Dentin etched and rinsed as in Group 1 and gently air dried for 5 s;
- Group 5: Dentin etched and pretreated as in Group 1, followed by the application of a universal adhesive system (Futurabond M, Voco, Cuxhaven, Germany) according to the manufacturer’s instructions and polymerized for 10 s using a LED curing unit (Demi, Kerr, Germany) after which a 1 mm thick layer of flowable composite was applied to the bonded surface (Grandio Flow, Voco, Cuxhaven, Germany) and polymerized for 20 s;
- Group 6: Dentin etched and pretreated as in Group 2, followed by the adhesive and restorative procedures as in Group 5;
- Group 7: Dentin etched and pretreated as in Group 3, followed by the adhesive and restorative procedures as in Group 5;
- Group 8 (control): Dentin etched as in Group 4, followed by the adhesive and restorative procedures as in Group 5.

After 24 h storage in the artificial saliva on 37 °C, the dentin specimens were cut into 1 mm thick sticks, glued to glass slides (2 sticks per tooth per group), ground down to approximately 50 µm thickness and subjected to the in situ zymography protocol by Mazzoni et al. [6,42]. Briefly, the samples were covered in diluted fluorescein-quenched gelatin, protected with a glass coverslip and kept in a dark humid chamber overnight at 37 °C, after which the specimens were observed by using a confocal microscope (Leica SP8, Leica Microsystems GmbH, Wetzlar, Germany; excitation/emission wavelength: 488/530 nm). Three images (z-stack, one image after every 1 µm into the depth of the sample) were made for each stick on randomly chosen sites by a blinded operator. Depending on the group, either the pretreated dentin surfaces or the hybrid layers were captured on the images. The integrated density of the fluorescent signal was further measured on all the images using the ImageJ software (National Institutes of Health, Bethesda, MD, USA), and the data were statistically analyzed using SigmaPlot 14.0 (Systat Software Inc., Berkshire, UK). As the data passed the assumptions of normal distribution and homogeneity, two-way ANOVA test was used for the analysis (factors “pretreatment” and “adhesive system application”). The significance level was set at *p* < 0.05. 

## 5. Conclusions

Based on the results of the present study, treating demineralized dentin with 0.1% chitosan in water inhibited dentin endogenous proteases expression, whereas higher concentrations of chitosan showed intense enzymatic activity when used before the adhesive procedures.

## Data Availability

The data presented in this study are available on request from the corresponding author.
